# Computed Tomography-Derived Left Ventricular Extracellular Volume Predicts Reverse Remodeling After Catheter Ablation for Atrial Fibrillation

**DOI:** 10.3390/jcdd13060264

**Published:** 2026-06-11

**Authors:** Makiko Kinoshita, Hiroyuki Takaoka, Yusei Nishikawa, Yoshitada Noguchi, Katsuya Suzuki, Shuhei Aoki, Satomi Yashima, Kazuki Yoshida, Haruka Sasaki, Noriko Suzuki-Eguchi, Tomonori Kanaeda, Yusuke Kondo, Yoshio Kobayashi

**Affiliations:** 1 Department of Cardiovascular Medicine, Chiba University Graduate School of Medicine, Chiba 260-0856, Japan; 2 Department of Clinical Laboratory, The University of Tokyo Hospital, Tokyo 113-8655, Japan; 3 Department of Cardiovascular Medicine, Eastern Chiba Medical Center, Chiba 283-8686, Japan

**Keywords:** computed tomography, extracellular volume, atrial fibrillation, catheter ablation, reverse remodeling

## Abstract

Left ventricular (LV) extracellular volume fraction (ECV) quantified by cardiac computed tomography (CT) reflects diffuse myocardial fibrosis. In patients with atrial fibrillation (AF) and reduced LV ejection fraction (LVEF), distinguishing tachycardia-induced cardiomyopathy from underlying myocardial disease remains challenging. The prognostic value of ECV for predicting reverse remodeling (RR) after catheter ablation for AF remains uncertain. We retrospectively analyzed 102 patients with LVEF ≤ 50% on echocardiography who underwent cardiac CT before AF ablation between May 2015 and April 2025. RR was defined as a ≥15% reduction in LV end-systolic volume with recovery of LVEF > 50%, or an absolute increase in LVEF of ≥15%. RR occurred in 49 patients (48%). ECV was significantly lower in patients with RR than in those without (31.2 ± 3.5% vs. 37.6 ± 7.8%, *p* < 0.001). Receiver operating characteristic analysis identified an optimal cutoff of 34.8% (area under the curve 0.77; sensitivity 88%; specificity 62%, *p* < 0.001). In multivariable logistic regression analysis, lower ECV remained independently associated with RR (odds ratio 0.84; 95% confidence interval 0.75–0.95; *p* = 0.006). CT-derived ECV was associated with RR after AF ablation in patients with reduced LVEF and may provide additional information for clinical decision-making.

## 1. Introduction

Catheter ablation is an established treatment option for many patients with atrial fibrillation (AF), and reverse remodeling (RR) of the left ventricle (LV) is frequently observed after ablation in patients with LV dysfunction [1]. However, reliable predictors of RR remain elusive.

Cardiac magnetic resonance (CMR) with late gadolinium enhancement (LGE) and T1 mapping enables assessment of focal and diffuse myocardial fibrosis [2,3]. However, CMR may be limited in patients with arrhythmia, implanted devices, or advanced renal dysfunction.

Cardiac computed tomography (CT) is widely used for coronary artery evaluation [4]. Recent technological advances in CT allow high-quality imaging even in patients with AF [5]. Late-phase cardiac CT can detect myocardial injury as late iodine enhancement and intracardiac thrombus [6] and allows quantification of LV ECV comparable to CMR [7,8].

We hypothesized that CT-derived ECV could predict RR after AF ablation in patients with LV dysfunction. In a prior small cohort, we were unable to demonstrate the predictive value of ECV alone. In the present study, with a larger population, we reassessed whether CT-derived ECV independently predicts RR after AF ablation [9].

## 2. Materials and Methods

### 2.1. Study Population

**Figure 1:**
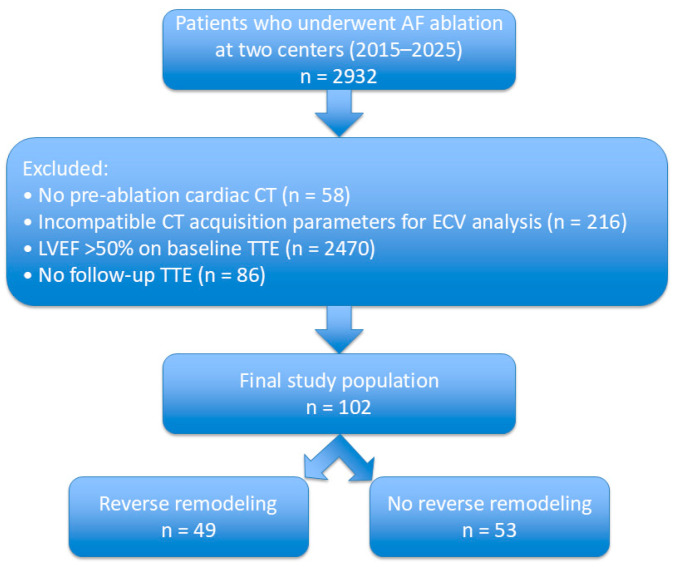
Flow diagram of patient selection and stepwise exclusions. The diagram illustrates the inclusion and exclusion process leading to the final study population of 102 patients.

This retrospective study included 102 consecutive patients with AF and LVEF ≤ 50% on transthoracic echocardiography (TTE), who underwent cardiac CT including a late-phase scan, before catheter ablation for AF between May 2015 and April 2025 at Chiba University Hospital (Institution 1) and Eastern Chiba Medical Center (Institution 2). All patients underwent both pre- and post-ablation TTE. The patient selection process is illustrated in Figure 1. Some patients were excluded because mismatched tube voltage settings between early- and late-phase acquisitions precluded reliable ECV calculation.

RR was defined as ≥15% reduction in LV end-systolic volume (LVESV) with recovery of LVEF > 50%, or an absolute increase in LVEF ≥ 15% [10,11]. The institutional review boards of both hospitals approved this study and waived the requirement for informed consent because of its retrospective design.

AF subtype was classified according to contemporary guideline definitions as paroxysmal AF (self-terminating within 7 days) or persistent AF (sustained beyond 7 days or requiring cardioversion), with long-standing persistent AF included in the persistent AF group. Follow-up evaluation included outpatient clinical visits with 12-lead electrocardiography, and additional Holter monitoring was performed at the discretion of the treating physician or when arrhythmia recurrence was suspected based on symptoms. Early AF recurrence was defined as any documented atrial arrhythmia lasting ≥30 s occurring within the 3-month blanking period after ablation, whereas AF recurrence was defined as any documented atrial arrhythmia lasting ≥30 s after the 3-month blanking period. Prior heart failure hospitalization was defined as a documented hospitalization for heart failure in the medical records.

### 2.2. CT Acquisition Protocol

Cardiac CT was performed using either a 256-row multidetector scanner (Revolution CT Apex; GE HealthCare, Waukesha, WI, USA) or a 320-row multidetector scanner (Aquilion ONE/ViSION Edition; Canon Medical Systems, Otawara, Japan). After a scout scan, a non-contrast electrocardiogram (ECG)-gated acquisition was obtained. The 320-row CT used slice thickness of 0.5 mm with tube voltage of 80–120 kV, whereas the 256-row CT used slice thickness of 0.625 mm and tube voltage of 70–120 kV, and tube current was automatically adjusted by the automatic exposure control (AEC) system. For the early contrast-enhanced scan, retrospective ECG gating with dose modulation was applied [12]. Patients with a heart rate ≥ 65 bpm received either 20 mg of metoprolol tartrate orally or 12.5 mg of landiolol intravenously, unless contraindicated. Contrast medium (50–110 mL, iodine concentration 300–370 mg/mL) was injected through an antecubital vein at 3–5 mL/s, followed by a 50% contrast–50% saline mixture and a saline flush [13]. During the earlier part of the study period, a fixed contrast volume of approximately 95 mL was typically used, with adjustments based on body habitus or renal function. In the current protocol of Institution 1, contrast dose is generally administered according to body weight (600 mgI/kg). Late-phase imaging was performed 6 min after contrast injection using prospective ECG gating [14], routinely for evaluation of left atrial thrombus. Prospectively ECG-gated images were generally reconstructed during the diastolic phase, typically around 75% of the RR interval, although the optimal phase was individually selected according to motion artifact in patients with tachycardia or AF. Slice thickness was 0.5 mm, tube voltage was set to 100 or 120 kV (same as the non-contrast acquisition), and tube current was again determined by the AEC system.

### 2.3. CT-Derived ECV Analysis

**Figure 2:**
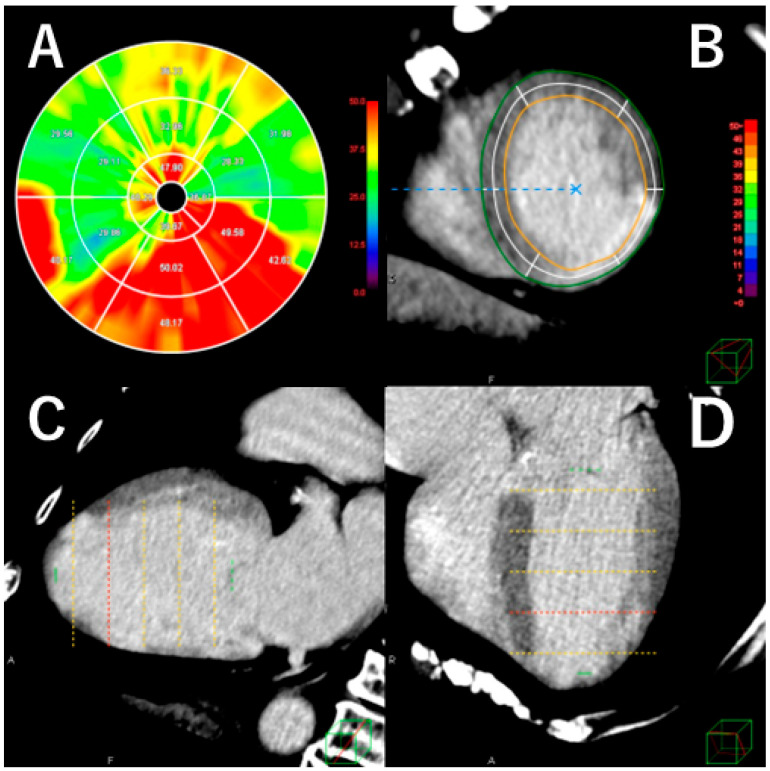
Left ventricular extracellular volume fraction (ECV) analysis on Computed tomography (CT). CT images in a 76-year-old female without reverse remodeling. Regional ECV elevation corresponds to a prior myocardial infarction related to atrial fibrillation–associated coronary embolism. ECV was calculated as the mean value across the 16 left ventricular segments (**A**). Quantitative ECV analysis was performed using short-axis, two-chamber, and four-chamber views (**B**–**D**). In (**C**,**D**), the colored dotted lines indicate the locations of short-axis slices used for ECV analysis, and the red dotted line corresponds to the slice displayed in (**B**).

LV ECV was quantified using commercial software (Ziostation 2; Ziosoft Inc., Tokyo, Japan) according to the following equation:

ECV (%) = (1 − hematocrit) × ΔHUm/ΔHUb × 100

where hematocrit was entered as a fractional value, and ΔHUm and ΔHUb represent the changes in myocardial and blood attenuation in Hounsfield units between non-contrast and late-phase scans [14]. For blood pool measurement, regions of interest were placed in the mid-ventricular LV cavity. Subtraction images were generated by automatic three-dimensional (3D) non-rigid registration using short-axis, two-and four-chamber views (Figure 2B–D). Polar maps of the 16 American Heart Association LV segments were automatically created, and the mean ECV was calculated across all segments (Figure 2A). Regions of interest were manually adjusted if misregistration was suspected. Interobserver reproducibility was evaluated in 20 randomly selected patients by two independent readers.

### 2.4. Ablation Procedure

Pulmonary vein isolation was performed using a 3D electroanatomic mapping system (Carto3; Biosense Webster, Diamond Bar, CA, USA) merged with CT images (CartoMerge; Biosense Webster). An 8.4-Fr irrigated-tip catheter was used for mapping and ablation [15]. Radiofrequency energy was delivered at 30–35 W with a temperature limit of 42 °C. Ipsilateral pulmonary veins were circumferentially isolated, and additional lines (roof line, posterior wall, mitral isthmus, or cavotricuspid isthmus) were created at operator’s discretion. Intraprocedural anticoagulation was maintained with activated clotting time > 300 s. Early recurrence of AF within 3 months and late recurrence thereafter were defined according to consensus criteria [16,17].

### 2.5. Statistical Analysis

Continuous variables are expressed as mean ± standard deviation (SD) or median (interquartile range [IQR]) as appropriate, and categorical variables as counts and percentages. Group comparisons were performed using Student’s *t*-test for continuous variables and Fisher’s exact test for categorical variables.

To identify independent predictors of RR, multivariable logistic regression analyses were performed. Given the limited number of events, the number of variables was restricted to maintain an events-per-variable ratio of approximately 5–10 to avoid overfitting [18,19]. Variables included in the model were selected based on univariable analysis results and clinical relevance.

Sensitivity analyses were performed excluding patients with cardiomyopathy (dilated cardiomyopathy, cardiac amyloidosis, cardiac sarcoidosis, hypertrophic cardiomyopathy and cancer therapeutics-related cardiomyopathy) or prior myocardial infarction (MI) to evaluate the robustness of the association between CT-derived ECV and RR.

Independent predictors were expressed as odds ratios (ORs) with 95% confidence intervals (CIs). ROC analysis was performed to determine the optimal ECV cut-off value using the Youden index. Interobserver agreement was assessed using the intraclass correlation coefficient (ICC) and Pearson correlation coefficient. All statistical analyses were performed using EZR (Version 1.68; Jichi Medical University, Saitama, Japan), a graphical user interface for R.

## 3. Results

LV RR was achieved in 49 patients (48%) at follow-up echocardiography performed a median of 8.9 months after ablation.

### 3.1. Baseline Characteristics

Baseline characteristics of patients with and without RR are summarized in Table 1. Patients who achieved RR were significantly younger (62 ± 10 vs. 67 ± 11 years, *p* = 0.036) and had a lower prevalence of DM (10% vs. 32%, *p* = 0.008) and cardiomyopathy (4% vs. 30%, *p* < 0.001). There were no notable differences in body mass index, hypertension, renal function, or AF duration between the two groups.

### 3.2. Imaging Findings

Echocardiographic parameters such as baseline LVEF and LV volumes were similar between groups. However, CT-derived ECV was markedly lower in the RR group compared with the non-RR group (31.2 ± 3.5% vs. 37.6 ± 7.8%, *p* < 0.001). Late iodine enhancement was also more common in patients without RR (53% vs. 26%, *p* = 0.002) (Table 2). The mean iodine load was 532 ± 118 mgI/kg.

### 3.3. Medications and Ablation Procedures

Patients with RR were less frequently treated with statins (20% vs. 43%, *p* = 0.012), mineralocorticoid receptor antagonists (MRA) (33% vs. 55%, *p* = 0.027) and sodium-glucose co-transporter 2 inhibitors (SGLT2i) (12% vs. 53%, *p* < 0.001), although there were no significant differences in ablation procedures between the two groups (Table 3).

### 3.4. Predictors of Reverse Remodeling

**Figure 3:**
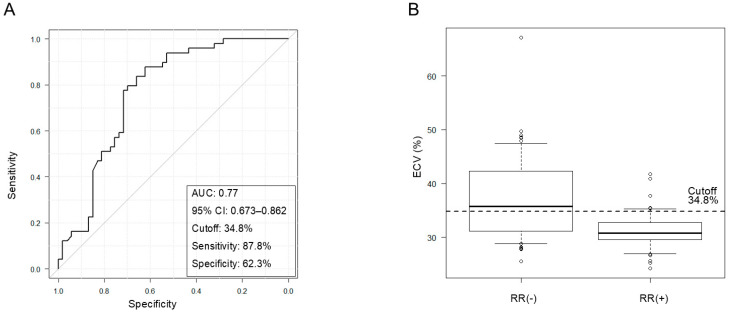
(**A**) Receiver operating characteristics (ROC) curve of left ventricular extracellular volume fraction (ECV) for predicting reverse remodeling (RR) after atrial fibrillation (AF) ablation. The optimal cutoff value of ECV was 34.8%, with a sensitivity of 87.8% and a specificity of 62.3% (AUC = 0.77, 95% CI 0.673–0.862, *p* < 0.001). (**B**) Distribution of CT-derived LV ECV according to RR status. Box-and-whisker plots showing CT-derived LV ECV values in patients with (RR+) and without (RR−). The median, interquartile range, and individual data points are presented. A horizontal dashed line indicates the cutoff value (ECV = 34.8%).

In multivariable logistic regression analysis, lower ECV was an independent predictor of RR (*p* < 0.01; Table 4). ROC analysis identified an optimal ECV cut-off of 34.8%, with a sensitivity of 87.8% and a specificity of 62.3% (AUC = 0.77, 95% CI 0.673–0.862, *p* < 0.001; Figure 3). Model comparison using Akaike’s Information Criterion showed a numerically lower AIC after inclusion of ECV (AIC 80.7 vs. 83.4). The corresponding base model included age, DM, cardiomyopathy, late iodine enhancement, statin use, and MRA use.

After exclusion of patients with cardiomyopathy (classified as described in the Methods; *n* = 17), ECV remained significantly lower in cases with RR than in those without RR (31 ± 3% vs. 35 ± 6%, *p* < 0.001). Similarly, after exclusion of patients with prior myocardial infarction (*n* = 16), ECV remained significantly lower in patients with RR (31 ± 4% vs. 37 ± 8%, *p* < 0.001).

ECV showed a moderate inverse correlation with change in LVEF (r = −0.416) and a weak inverse correlation with LVESV reduction (r = −0.297), supporting the association between higher myocardial fibrosis burden and less favorable ventricular recovery after AF ablation.

In contrast, ECV did not differ significantly between patients with and without AF recurrence (33 ± 6% vs. 35 ± 7%, *p* = 0.20).

Follow-up echocardiography was performed at a median of 8.9 months (IQR 4-11.6). In a sensitivity analysis restricted to 38 patients who underwent echocardiography between 6 and 12 months, ECV remained significantly lower in cases with RR than in those without RR (31 ± 3% vs. 38 ± 6%, *p* < 0.001).

### 3.5. Reproducibility and Safety

Interobserver correlation for ECV measured by two independent observers in 20 randomly selected patients was excellent (ICC = 0.87; 95% CI, 0.689–0.951; Pearson r = 0.97, *p* < 0.001). No major thromboembolic events occurred after ablation.

## 4. Discussion

This study demonstrated that CT-derived LV ECV was an independent predictor of RR after catheter ablation in patients with AF and reduced LVEF. Patients who achieved RR exhibited significantly lower ECV values, suggesting a lower degree of myocardial fibrosis and a higher potential for functional recovery. These findings indicate that CT-based myocardial tissue characterization provides not only anatomic but also prognostic information in AF patients undergoing ablation.

### 4.1. CT-Derived ECV and Myocardial Fibrosis

AF accompanied by reduced LV function may result from either tachycardia-induced cardiomyopathy (TIC) or structural myocardial disease, and differentiating these entities is important for treatment decisions. The present finding that lower ECV was associated with RR may be consistent with the concept of TIC, in which LV dysfunction is potentially reversible after rhythm control [20,21,22]. The definition of RR used in the present study incorporated both improvement in LV systolic function and reduction in LV volume, aiming to reflect clinically meaningful ventricular recovery after ablation. Patients without RR showed higher ECV values, suggesting more advanced myocardial remodeling and less reversible myocardial damage.

Sensitivity analyses confirmed that the prognostic value of ECV was not driven by specific cardiomyopathy subtypes but reflected underlying myocardial tissue characteristics related to reversibility. Accordingly, CT-derived ECV may serve as a noninvasive biomarker to help distinguish reversible from irreversible LV dysfunction in patients with AF. However, the predictive performance of ECV for RR was moderate (AUC = 0.77), likely reflecting the multifactorial mechanisms underlying LV recovery after AF ablation. Future studies may improve prediction by integrating ECV with additional clinical or imaging variables.

Previous studies using CMR have also demonstrated that increased ECV is associated with less favorable LV functional recovery and cardiac remodeling after AF ablation [23,24]. These findings support the concept that myocardial ECV reflects the degree of irreversible myocardial remodeling regardless of the imaging modality.

In addition, recent advances in delayed-iodine CT imaging enable myocardial tissue characterization analogous to late gadolinium enhancement on CMR. Given the heterogeneous myocardial substrates associated with AF, CT-based tissue characterization may support more individualized management.

### 4.2. Clinical Value and Technical Feasibility of CT

CMR remains the gold standard for assessing myocardial fibrosis [25], but it is limited by contraindications, long scan times, and arrhythmia-related artifacts [4]. CT is widely available, has a shorter acquisition time, and is feasible in patients with AF [5,6]. Beyond anatomical evaluation, late-phase CT allows quantification of myocardial fibrosis through ECV analysis and has shown strong correlation with CMR-derived measurements in previous studies [7,8].

Our results support the role of CT-derived ECV as a prognostic imaging biomarker [26]. Importantly, CT-derived ECV analysis has been included in the latest Japanese Circulation Society guideline for cardiac amyloidosis as an alternative to CMR [27]. A report indicated that amyloid deposition was observed in 12 cases (5%) out of 230 cases undergoing atrial biopsy for AF ablation. CT delayed imaging may be useful for early detection and therapeutic decision-making [28].

### 4.3. Radiation Exposure and Technical Considerations

A potential limitation of CT-based myocardial evaluation is additional radiation exposure. However, in our previous experience, the radiation dose for late-phase acquisition (mean 5.4 mSv) was comparable to that of standard chest CT [9,29,30]. Advances in CT technology, including deep-learning reconstruction, have improved image quality while reducing radiation dose [6,7]. In our protocol, prospective ECG gating was employed to minimize additional exposure without compromising diagnostic accuracy. Detailed radiation dose metrics for the late-phase acquisition were available in a subset of 11 patients, in whom the additional computed tomography dose index (CTDI) volume was 33.9 ± 15.1 mGy, which was comparable to that of a standard cardiac CT acquisition.

### 4.4. SGLT2 Inhibitors and Reverse Remodeling

In a sensitivity analysis replacing diabetes mellitus with SGLT2 inhibitor use, both CT-derived ECV (*p* = 0.01) and SGLT2 inhibitor use (*p* = 0.046) were independently associated with reverse remodeling.

Interestingly, patients receiving SGLT2i were less likely to achieve RR in our cohort. This paradox may reflect confounding by indication, as SGLT2i users tended to have more advanced comorbidities and higher baseline fibrosis. In addition, the beneficial effects of SGLT2i on remodeling may require longer follow-up periods to become evident [31,32,33]. Furthermore, the use of SGLT2i has only recently been approved and implemented in clinical practice in Japan, which may have influenced our results. Therefore, the absence of RR in SGLT2i users in our study should be interpreted cautiously.

### 4.5. Clinical Implications

Our findings suggest that CT-derived ECV can provide additional value in the pre-ablation evaluation. Because cardiac CT is already routinely performed for anatomical assessment, extending the protocol to include late-phase imaging may provide prognostic information without requiring other imaging modalities [34,35]. Early identification of cardiomyopathies such as cardiac amyloidosis may also enable timely disease-specific treatment. CT-derived ECV may therefore support improved patient selection and more individualized treatment strategies.

## 5. Limitations

This study has several limitations. First, this was a retrospective two-center analysis, which may limit the generalizability, and the long enrollment period may have introduced temporal bias, although CT acquisition and analysis protocols were standardized. Second, the study population was reduced by the requirement for both late-phase CT imaging and follow-up echocardiography, and incomplete follow-up in referred patients may have introduced selection bias. Third, not all patients received full guideline-directed medical therapy before ablation, and post-ablation medication changes were not systematically available. Fourth, variability in follow-up intervals may have affected the assessment of RR, as ventricular recovery after ablation would be time-dependent. Fifth, systematic validation with CMR and serial imaging was not available due to real-world clinical constraints. Sixth, clinical outcome data after follow-up echocardiography were not consistently available, precluding assessment of the relationship between reverse remodeling and subsequent clinical outcomes. Finally, some patients may have been scanned during AF rhythm, which could have introduced misregistration and affected ECV measurement. In addition, the optimal ECV cut-off was data-driven and was not validated in an external cohort, requiring further validation in independent cohorts.

## 6. Conclusions

CT-derived LV ECV independently predicts reverse remodeling after AF ablation. Incorporating ECV analysis into routine pre-ablation CT could provide additional prognostic information beyond anatomical assessment, thereby contributing to improved patient selection and clinical management.

## Figures and Tables

**Table 1:** Baseline characteristics of patients with and without reverse remodeling after catheter ablation.

	RR (+) (*n* = 49)	RR (−) (*n* = 53)	*p* Value
Age, y	62 ± 10	67 ± 11	0.036
Male, *n* (%)	36 (73%)	38 (72%)	1
Body mass index, kg/m^2^	24.1 ± 3.5	23.8 ± 4.6	0.751
Hypertension, *n* (%)	29 (59%)	27 (51%)	0.549
Dyslipidemia, *n* (%)	11 (22%)	21 (40%)	0.058
Diabetes mellitus, *n* (%)	5 (10%)	17 (32%)	0.008
NYHA class	2.0 ± 1.1	2.0 ± 1.0	0.995
BNP, pg/mL	160 (20–886)	191 (11–1941)	0.249
Hematocrit, %	43.3 ± 4.6	43.0 ± 4.5	0.739
eGFR, mL/min/1.73 m^2^	59.7 ± 16.4	54.2 ± 14.6	0.075
Duration of AF, months	15 ± 19	15 ± 26	0.987
Persistent AF, *n* (%)	34 (69%)	30 (57%)	0.302
CHADS2 score	1.8 ± 1.1	2.1 ± 1.1	0.206
Early AF recurrence, *n* (%)	13 (27%)	19 (36%)	0.295
AF recurrence, *n* (%)	14 (29%)	13 (25%)	0.659
Smoking, *n* (%)	26 (53%)	33 (62%)	0.318
Family history of CAD, *n* (%)	11 (26%)	11 (21%)	1
Prior HF hospitalization, *n* (%)	25 (51%)	28 (53%)	0.843
Cardiomyopathy, *n* (%)	2 (4%)	15 (28%)	0.001

RR: reverse remodeling, AF: atrial fibrillation, CAD: coronary artery disease, HF: heart failure.

**Table 2:** TTE and CT parameters in patients with and without reverse remodeling.

	RR (+) (*n* = 49)	RR (−) (*n* = 53)	*p* Value
LVEF, %	36.5 ± 9.0	37.0 ± 8.3	0.804
LA diameter, mm	44.4 ± 6.1	43.4 ± 5.5	0.412
LVDd, mm	53.1 ± 7.6	54.2 ± 7.6	0.483
LVDs, mm	43.7 ± 8.1	44.3 ± 9.0	0.745
LVEDV, mL	134.4 ± 49.5	136.7 ± 60.0	0.834
LVESV, mL	87.0 ± 43.9	89.3 ± 48.6	0.807
Interval between TTEs, months	10.3 ± 8.3	7.5 ± 5.9	0.055
Significant coronary stenosis, *n* (%)	11 (26%)	11 (21%)	1
Late iodine enhancement, *n* (%)	11 (26%)	28 (53%)	0.002
ECV on CT, %	31.2 ± 3.5	37.6 ± 7.8	<0.001
Post LVEF, %	57.8 ± 6.5	42.8 ± 12.9	<0.001
Post LVEDV, mL	113.3 ± 31.5	145.0 ± 65.1	0.003
Post LVESV, mL	49.1 ± 20.8	89.6 ± 53.8	<0.001

TTE: transthoracic echocardiography, CT: computed tomography, LVEF: left ventricular ejection fraction, LA: left atrium, LVEDV: left ventricular end-diastolic volume, LVESV: left ventricular end-systolic volume, ECV: extracellular volume fraction.

**Table 3:** Baseline medications and catheter ablation procedures in patients with and without reverse remodeling.

	RR (+) (*n* = 49)	RR (−) (*n* = 53)	*p* Value
β blocker, *n* (%)	41 (84%)	45 (85%)	0.783
Amiodarone, *n* (%)	6 (12%)	9 (17%)	0.58
Statin, *n* (%)	10 (20%)	23 (43%)	0.012
ACEi/ARB/ARNI, *n* (%)	31 (63%)	40 (75%)	0.202
MRA, *n* (%)	16 (33%)	29 (55%)	0.027
SGLT2i, *n* (%)	6 (12%)	28 (53%)	<0.001
Pulmonary vein isolation, *n* (%)	49 (100%)	51 (96%)	0.496
Left atrial posterior wall isolation, *n* (%)	12 (24%)	9 (17%)	0.337
Roof line isolation, *n* (%)	14 (29%)	11 (21%)	0.368
Left mitral isthmus line isolation, *n* (%)	10 (20%)	10 (19%)	1
Cavotricuspid isthmus line isolation, *n* (%)	25 (51%)	30 (57%)	0.688

ACEi: angiotensin converting enzyme inhibitor, ARB: angiotensin receptor II blocker, ARNI: angiotensin receptor blocker and neprilysin inhibitor, MRA: mineralocorticoid receptor antagonist, SGLT2i: sodium-glucose co-transporter 2 inhibitor.

**Table 4:** Univariate and multivariate logistic regression analysis of predictors of reverse remodeling.

	Univariate		Multivariate	
	OR (95% CI)	*p* Value	OR (95% CI)	*p* Value
Age	0.96 (0.92–1.00)	0.04	0.98 (0.94–1.03)	0.39
Diabetes Mellitus	0.26 (0.09–0.77)	0.015	0.47 (0.13–1.74)	0.26
Cardiomyopathy	0.10 (0.02–0.44)	<0.01	0.45 (0.07–2.83)	0.39
Late iodine enhancement	0.25 (0.11–0.59)	<0.01	0.90 (0.27–2.95)	0.86
ECV on CT	0.80 (0.72–0.89)	<0.001	0.84 (0.75–0.95)	<0.01
Statin	0.32 (0.13–0.78)	0.012	0.61 (0.20–1.81)	0.37
MRA	0.39 (0.17–0.86)	0.02	0.91 (0.33–2.55)	0.86
SGLT2i	0.11 (0.04–0.31)	<0.001		

OR: odds ratio, CI: confidence intervals, MRA: mineralocorticoid receptor antagonist, SGLT2i: sodium-glucose co-transporter 2 inhibitor

## Data Availability

The original contributions presented in this study are included in the article. Further inquiries can be directed to the corresponding author.
